# Statistical Models Supporting the High-Performance Self-Compacting Concrete (HPSCC) Design Process for High Strength

**DOI:** 10.3390/ma15020690

**Published:** 2022-01-17

**Authors:** Aleksandra Kostrzanowska-Siedlarz, Jacek Gołaszewski

**Affiliations:** Faculty of Civil Engineering, Silesian University of Technology, 44-100 Gliwice, Poland; jacek.golaszewski@polsl.pl

**Keywords:** ANOVA, compressive strength, high-performance self-compacting concrete, HPSCC

## Abstract

The type of test ingredients used for obtaining self-compacting high-performance concrete (HPSCC) has been carefully selected to be universal. For this purpose, an extensive statistical analysis of the obtained results of the literature research was carried out. Then, universal and adapted to the typical range, highly fit statistical models are presented that can support the HPSCC design process for achieving high strength. For this purpose, a broad plan of statistical research was used, namely multivariate selection of sidereal points, which allowed the use of as many as five variable factors at three levels of variability. The sidereal points were equal to the respective minimum and maximum input values. Additionally, based on the analysis of variance (ANOVA) for factorial systems with the interaction of the obtained test results, the significance of the impact of the tested material factors on the compressive strength of the HPSCC tested was determined.

## 1. Introduction

High-performance self-compacting concrete (HPSCC) combines the features of a mixture of self-compacting concrete (SCC) and hardened high-performance concrete (HPC). HPC technology was initially created at the turn of the 1970s. The initial goal of HPC was to obtain concrete with compressive strength of at least 60 MPa (f_ck,cub_ ≥ 60 MPa, f_ck,cyl_ ≥ 50 MPa) [1,2,3,4,5]. However, SCC was first designed in 1988 in Japan. The immediate reason for undertaking work on the development of SCC was the need to eliminate the vibration process [6,7,8,9,10,11]. SCC technology has increased the concreting efficiency, reduced labor costs, and increased the quality of concrete after hardening. Less than 10 years later, the first attempts to combine the two SCC and HPC technologies were also made in Japan, which resulted in the creation of HPSCC [12,13,14,15]. HPSCC is a cement-aggregate composite that is designed for obtaining compressive strength greater than 60 MPa whose components are also selected for the specific properties of the concrete mix. These properties should ensure the ability of the mix to tightly fill the space between the reinforcement and the space inside the formwork under the influence of its own weight without the need for mechanical compaction [12,13,14,15,16,17,18,19,20]. The use of HPSCC gives the possibility of concreting elements with dense reinforcement and complex shapes without the need for vibrations, as well as obtaining a very accurate mapping of mold surfaces without surface defects (architectural concrete). At the same time, it eliminates a number of disadvantages associated with vibratory compaction, e.g., noise and vibration, and reduces the work involved in forming elements. Currently, highly developed concrete technology allows the requirements to be specified for both the set of features of hardened concrete and the properties of the concrete mix, depending on the design requirements. The changes taking place in this field set new directions for the modification of properties of concrete mix and hardened concrete properties. This leads to high-quality concrete.

HPSCC must comply with self-compacting conditions (appropriate rheological properties and the appropriate amount of air in the mixture), but also achieve high compressive strength. The next sub-section of the article shows how high compressive strength is obtained after 28 days. Based on the literature, it was shown what else should be tested in this regard in order to facilitate the HPSCC design process due to its high strength. 

For this purpose, a broad plan of statistical research was used, including multivariate selection of sidereal points, which allowed the use of as many as five variable factors at three levels of variability. The sidereal points were equal to the respective minimum and maximum input values. Additionally, based on the analysis of variance for factorial systems with the interaction of the obtained test results, the significance of the impact of the tested material factors on the compressive strength of the HPSCC tested was determined.

The last part of the article describes an additional aspect of the research, which was to compare the rheological and strength results with each other.

### 1.1. Analysis of HPSCC Compressive Strength and Composition—Case Review

For the purposes of developing data from literature, the STATISTICA computer program used (producer StatSoft Europe GmbH). For the purposes of the literature analysis, the following statistical tools were used:

The histogram is a graphical representation of the empirical distribution of 28-day compressive strength for HPSCC and HPC. This histogram shows the abundance percentages of the respective strengths for HPSCC and HPC, not the probability density, so the widths of the intervals are equal.

Analysis of variance for significance tests of factors influence: w/b ratio, percentage of condensed silica fume (CSF), amount of binder, aggregate, cement, and sand for compressive strength after 28 days. The purpose of analysis of variance is to test the significance of differences between means by comparing the variance.

The significance level (*p*-level) (usually, denoted by the symbol α). This corresponds to the probability of making a mistake in assuming the obtained result as true, i.e., representative for the population. The choice of α values depends on the researcher, the nature of the problem, and how accurately the hypotheses are verified. In many areas of research, the significance level of 0.05 is taken as the limit value of the acceptable error rate.

In the case of the F test (Value F), to evaluate the statistical significance of differences between the means, we calculate the quotient of the intergroup variance to the error variance. If the error variance decreases according to the explanatory power of the factor, then the global value F increases.

### 1.2. HPSCC Compressive Strength

**Figure 1:**
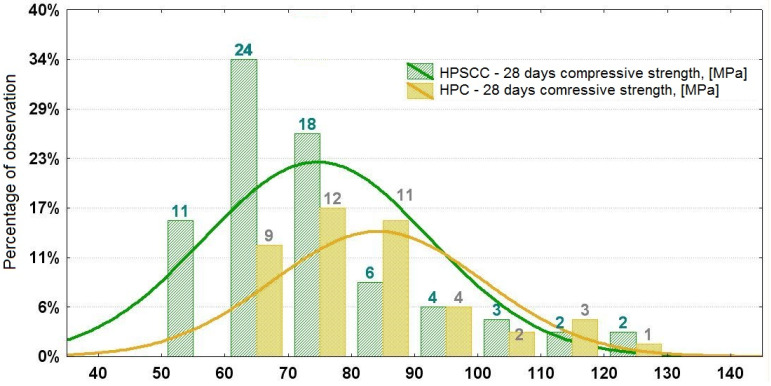
Histogram of 28-day compressive strength for HPSCC and HPC [18,21,22,23,24,25,26,27,28,29,30,31,32,33,34,35,36,37,38,39,40,41,42,43,44,45,46,47,48,49,50,51,52,53,54].

Analysis of the literature regarding HPSCC [18,21,22,23,24,25,26,27,28,29,30,31,32,33,34,35,36,37,38,39,40,41,42,43,44,45,46,47,48,49,50,51,52,53,54] shows that the values of compressive strength after 28 days range from 55 to about 135 MPa. About 60% of the analyzed HPSCC cases achieve strength within 60 ÷ 80 MPa (Figure 1). The analysis of the significance of the impact of HPSCC composition on compressive strength cases in the literature (Table 1) shows that the strength is mainly shaped by the ratio w/b and the percentage of CSF in the binder mass.

The analysis showed that in 12% of cases HPSCC are concretes designed as ordinary SCCs with gravel aggregate, which, however, due to the specificity of the composition ratio, achieved compressive strength ≥60 MPa. Other cases in the analysis are concretes already designed as high-strength concretes. Compressive strength is relatively unintentionally often obtained at 60 ÷ 80 MPa, while higher strengths are rarely obtained.

One of the reasons for the rare use of HPSCC is the lack of experimental data on the impact of HPSCC composition factors on the rheological properties of the mix and hardened concrete. There is a lot of information in the literature regarding the qualitative impact of these factors, but there is a lack of research to determine the hierarchy of the impact of HPSCC composition factors and to determine the quantitative impact of these factors on the above properties. Therefore, the aim of this research is to find the optimal compositions and determine the hierarchy of the influence of the components based on the analysis of variance and to determine statistical models for compressive strength by means of multiple regression. The intention is that the models can aid in the design of the HPSCC. Due to the wide range of variability of the basic parameters of HPSCC composition, adjusted to the typical range, used in the research, both the dependencies and functions are general in nature and can be effectively used in aiding design.

### 1.3. HPSCC Composition

The necessity to fulfill the requirements of the SCC rheological behavior and mechanical properties of hardened HPC determines the specificity of the composition of HPSCC. For the purposes of analyzing the composition of concrete, the composition and components of HPSCC were compared with the compositions of SCC (published by Domone [55]) and the composition of HPC (analysis of 56 selected cases [56,57,58,59,60,61,62,63,64]). The composition of HPSCC was analyzed on the basis of 76 cases [18,21,22,23,24,25,26,27,28,29,30,31,32,33,34,35,36,37,38,39,40,41,42,43,44,45,46,47,48,49,50,51,52,53,54]. Table 2 presents medians and percentiles of the amount of components in the composition of SCC, HPC, and HPSCC.

The analysis shows that the most common type of cement in the HPSCC composition is Portland cement CEM I 42.5R. In most of the analyzed cases, HPSCC most often used between 350 and 500 kg/m^3^ of cement, as in the case of HPC. There are also cases (5%) of the use of 500 to 600 kg/m^3^ cement in the HPSCC composition [36,65]. The median amount of cement in the HPSCC composition is 400 kg/m^3^, and the average value is 395 kg/m^3^. These values are smaller than the median value of the cement quantity in the HPC composition of 432 kg/m^3^ and the average value of 434 kg/m^3^. It is widely believed that the necessary condition for obtaining a HPSCC with a compressive strength of at least 80 MPa and high durability of concrete is the addition of condensed silica fume. This is confirmed by the analysis [18,21,22,23,24,25,26,27,28,29,30,31,32,33,34,35,36,37,38,39,40,41,42,43,44,45,46,47,48,49,50,51,52,53,54]. The fine grain size and highly developed surface of condensed silica fume grains have a positive effect on the porosity and water permeability of concrete and contribute to the increase in their strength. The mode of action of condensed silica fume in concrete consists of three effects: filling the pores between cement particles, increasing strength due to pozzolanic reaction with calcium hydroxide, and improving the connection between aggregate and cement stone [14,66,67,68,69]. The maximum amount of condensed silica fume added is related to the specific surface area of the aggregate. The greater the specific surface of the aggregate, the greater the amount of condensed silica fume is required to seal the contact zone of paste—aggregate. The recommended amount of condensed silica fume addition to HPC concretes is from 3 to 11% of the binder mass [2]. Studies have shown that the optimal amount in the HPC composition fluctuates within 10% of condensed silica fume (CSF) [5,57,70,71,72,73,74,75,76]. An increase in the amount of condensed silica fume over 10% compared to cement does not cause a clear decrease in the porosity of concrete. This is confirmed by the results of strength and water-absorption tests [77]. In Table 3, statistics for aggregate SCC, HPC, and HPSCC are compiled. The analysis showed that the median maximum aggregate grain size in the HPSCC composition is 16 mm, and the median amount of coarse aggregate is 907 kg/m^3^.

Studies have shown that the amount of fraction up to 2 mm in aggregate should not significantly exceed 50%. In most cases (over 80%), the aggregate sand point for HPSCC is between 38% and 51%. The amount of added superplasticizer in the composition of the analyzed HPSCC is between 0.5 and 3% of the binder mass. The most commonly used superplasticizers are based on polycarboxylate ethers (61%), slightly less often on the basis of melanin and naphthalene-formaldehyde sulfonates (39%). No superplasticizer based on lignosulfonates has been reported due to their less effective dispersing action compared to others.

In the literature [18,19,21,52,78,79,80,81,82], a lot of attention is paid to VEA and VMA (Viscosity Enhancing Agent, Viscosity Modifying Agent) admixtures. However, in practice, only 8.9% of the analyzed HPSCC cases used a viscosity-increasing admixture. The addition of viscosity-increasing additives can have a negative impact on compressive strength, which is why these admixtures are dosed with great care in the case of HPSCC.

The composition of SCC and HPC is similar. Therefore, it is possible to design HPSCC, the composition of which allows one to meet both the requirements as to the appropriate rheological properties of the self-compacting mixture, as well as the characteristics of hardened concrete, which are typical for HPC.

In the case of HPC, the amount of cement paste, the amount of aggregate, and the quality of bonds between them (adhesion) should be appropriately shaped due to high strength and low permeability. It is therefore necessary to minimize the water–binder ratio (w/b) and to use appropriate mineral additives and dispersing chemical admixtures, as well as to select the appropriate aggregate and its grain size [83,84,85], which is consistent with the specifics of SCC design. However, the effect of uncontrolled air entrainment through fluidizing admixtures should be taken into account [86].

Table 4 shows the typical composition for HPSCC, used most often, as shown by literature analysis. Seventy-six compositions for HPSCC were analyzed.

Based on the analysis of the HPSCC composition in the literature [18,21,22,23,24,25,26,27,28,29,30,31,32,33,34,35,36,37,38,39,40,41,42,43,44,45,46,47,48,49,50,51,52,53,54], the universal composition of HPSCC was established in the studies at three levels of variability, which is discussed in the next section.

## 2. Methodology and Materials of Research

### 2.1. Variable Factors and Study Plan

The variable factors proposed in the research are presented in Table 5. The proposed composition of high-performance self-compacting concrete pertain to the typical set of the aforementioned concrete used in practice on the basis of statistical analysis of the effects of literature research. In terms of changes, the cement mix had to have adequate fluidity. Therefore, the it was understood that even the minimal addition of SP must be relatively high. The major criterion for selecting variables was obtaining a high variability in compressive strength.

Index φ is called the content of paste in the mixture and is defined as follows [53,87]:

(1) $$\varphi_{}=\frac{V_{}}{A\cdot P_{}}$$

where:

*V*—absolute volume of paste in the mixture, (m^3^);
*A*—weight of aggregates, (kg);
*P*—specific porosity of the poured loosely stack available for water, (m^3^/kg).

Sand point is called a percentage of the aggregate mass of particles with dimensions of 0.00 ÷ 2.0 mm (the sum of the percentages of sieve: 0.063, 0.125, 0.25, 0.5, 1) [87].

Multivariate selection of sidereal points was applied due to the purpose and scope of the research. The test plan allowed us to lower the large number of measuring points in comparison with the complete research plan and also reduce the volume of material used for study.

The study was divided for the five input variables (variable factors) with one block and twenty-seven layouts (Table 6). Sidereal points are equivalent to the corresponding values of the minimum and maximum input values. The degree of matching identified in studies of mathematical models was tested based on independent control measurements.

Generally, measurements for compressive strength were performed on several samples. The maximal and average relative standard deviation for compressive strength of concretes—6.8% and 5.1%, respectively.

### 2.2. Methodology of Statistical Analysis

For the purposes of the statistical analysis in the research, the following statistical methods and techniques for analyzing measurement results in STATISTICA software (version 13.3) were used:

Lilliefors test and Shapiro—Wolf test: the Lilliefors test and the Shapiro–Wilk test are used to verify the hypothesis that the difference between the examined variable distribution (empirical distribution) and the normal distribution (theoretical distribution) is irrelevant. The Lilliefors test is a correction of the Kolmogorov–Smirnov test in which the mean value (μ) and standard deviation (σ) for the sample population are not known.

Tests are used to verify the hypotheses:

1. H0: the distribution of the examined feature in the population is a normal distribution;
2. H1: the distribution of the studied trait in the population is different from the normal distribution;
3. The *p*-value determined on the basis of the test statistic is compared with the significance level α;
4. if *p* ≤ α ⇒ reject H0 assuming H1;
5. if *p* > α ⇒ there are no grounds to reject H0.

ANOVA analysis of variance. The study used the analysis of variance (ANOVA) for factorial systems with interaction to test the significance of the influence of variable factors.

The purpose of the analysis of variance (ANOVA) is to test the significance of differences between means by comparing (i.e., analyzing) the variance. The method consists of dividing the total variance into different sources (related to the effects occurring in the considered system), which makes it possible to compare the variance corresponding to the variability between groups (or treatments) with the variability within the groups. Assuming that the null hypothesis (that there is no mean difference between groups or treatments in the population) is true, we can expect that the variance estimated by the within-group variability should be approximately equal to the variance estimated by the intergroup variability. In developing the research results, multi-factor models were adopted; i.e., the influence of various factors was considered jointly, taking into account the main effects and interactions up to the given degree.

Contour plot: The test results are presented using contour plot. A contour plot is a projection of a surface onto a two-dimensional plane (the surface is fitted to the three-dimensional data). Fit area values (deposited on the Z axis) are represented by areas of different color in a two-dimensional scatter plot.

Profiles of approximated values: The approximation profile creates a separate equation for each output quantity, which is fitted to the measured response values (for a given output quantity). As soon as these equations are obtained, approximate values of the output quantities are calculated for any combination of the values of the input quantities. To facilitate the estimation of the credibility of the approximation, the confidence intervals for the approximate values are shown.

Confidence interval: The confidence interval shows the values of the expected (average) dependent variable (compressive strength). The confidence interval for the value of the predicted dependent variable determines the range of values within which (with a given probability) we expect the true value of the dependent variable for given values of the independent variables.

Polynomial (or otherwise called nonlinear linearized) regression calculates the relationship between a dependent variable and one or more independent variables that may appear at higher powers (square, third power, etc.). Polynomial regression is a common technique for studying the curvilinear relationship.

### 2.3. Testing Procedures

Concrete mixes were prepared in a forced concrete mixer with a volume of V = 50 dm^3^. The mixing time was 5 min; first, dry ingredients (cement, aggregate, and condensed silica fume) were mixed for 1 min, and then water with the superplasticizer was added. Before the second measurement, the mix was mixed for 3 min. The study of the compressive strength was performed in accordance with standards: PN-EN 12390-2: 2011 and PN-EN 12390-3:2011. Concrete mixture after the rheology test was placed into molds of dimensions 10 × 10 × 10 cm^3^. The specimens were covered with a PVC foil for the first 24 hours, and then the specimens were demolded. The compressive strength was determined on samples after 28 days of curing of concrete samples in water at 20 ± 3 °C.

Additionally, rheological properties of fresh concretes were tested using the slump flow test. During the test, the propagation time to a diameter of 50 cm (T (s)) and the maximum diameter (D (cm)) are measured. Slump flow test after 20 and 60 min from mixing ingredients was performed. Temperature of research was 20 ± 1 °C. The test also provides some information on the possible segregation, which is assessed visually by visual stability index VSI [85], where 0 means high stability, 1 means stability, 2 means instability, and 3 means high instability.

### 2.4. Materials and Cement Mixes

Portland cement CEM I 42.5R was used in the tests. The properties of the cement used are determined by the cement supplier (loss on ignition—2.0%; insoluble parts—0.4%; the specific surface area—383 m^2^/kg; changes in volume, Le Chatelier—0.0mm; beginning of setting time—175 min). The content of cement in the mixtures depends on the w/b ratio and the index φ. The content of cement in the mixtures ranged from 438.6 to 638.8 kg/m^3^.

**Figure 2:**
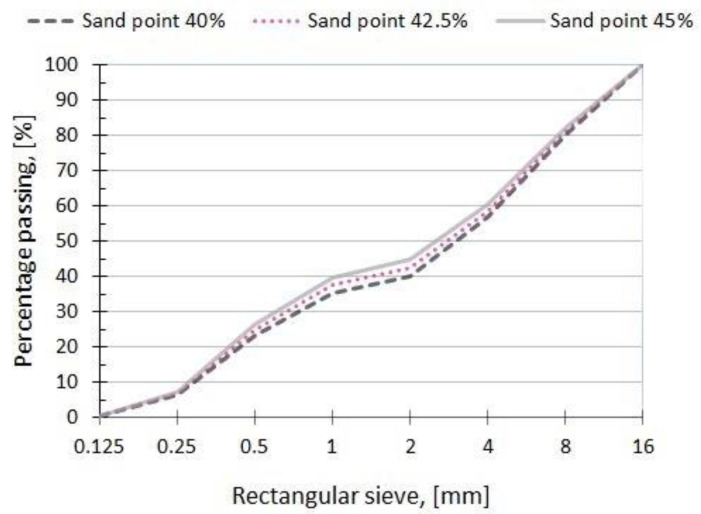
Grain size distribution of the aggregate with three sand points [87].

The aggregates used in this research are composed of natural sand 0–2 mm and syenite crushed aggregate in three fractions 2–4 mm, 4–8 mm and 8–16 mm, density of 2.62 kg/m^3^, 2.74 kg/m^3^ and 2.74 kg/m^3^, respectively. Grains with a size distribution of the aggregate with three sand points—40%, 42.5%, and 45%—were laid on the base according to recommendations in [19] (Figure 2).

The significant point influencing the rheological properties of the mix is also the type and content of superplasticizer (SP). In [86], authors showed that some superplasticizers can significantly and uncontrollably aerate the cement mix. The selection of superplasticizer compatible with the binder is important.

The choice of superplasticizer (SP) is compatible with condensed silica fume, and cement was made from twenty-four commercial superplasticizers. The chemical admixture selected as a SP with polyether base (density—1.09 g/cm^3^, concentration—34%). This is the most usually used type of admixture for Self-Compacting Concrete. The superplasticizer was dosed by weight and mixed with water.

The HPSCC composition does not contain VEA–VMA admixtures due to its possible negative impact on strength because of the effect of greater aeration of VMA and by impeding self-deaeration. This was described in the literature analysis in the Section 1.3.

The aim of choosing a suitable CSF was to search for compatibility with the cement and SP in research. From among the three CSFs from different suppliers, one was selected that was compatible with cement and SP used in the tests. The properties of the CSF used are determined by the supplier (the specific gravity (water = 1)—2.2; densities—350 kg/m^3^; the specific surface area—25 m^2^/g).

The content of condensed silica fume ranged from 0 to 62 kg/m^3^. 

## 3. Analysis of Test Results

**Figure 3:**
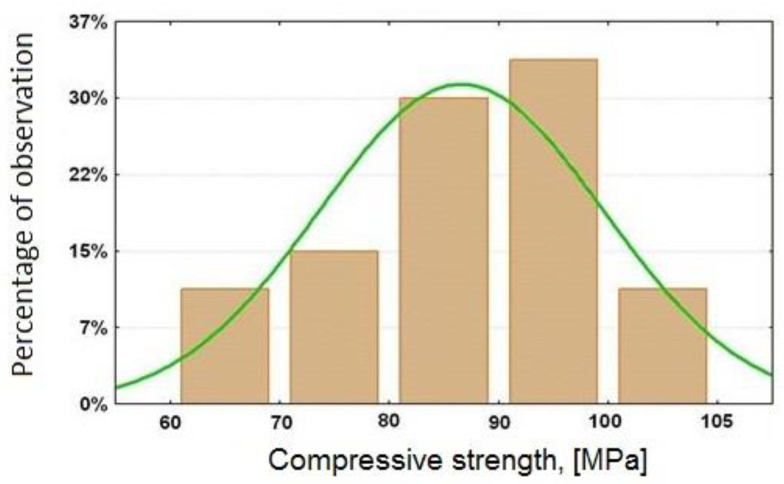
HPSCC compression strength histogram.

The tested concretes are characterized by compressive strength in the range of 60 MPa to 105.3 MPa (Table 7); therefore, concretes are classified into strength classes from C50/60 to C90/105, thus belonging to high strength concrete. In most of the tested concretes, the obtained compressive strength was in the range of 80 to 95 MPa (Figure 3). The self-compacting behavior was checked by the slump flow test, and the obtained results allowed for the qualification of the concretes to self-compacting concretes. The diameter of slump flow and the flow time were measured 20 and 60 min after mixing the ingredients.

**Figure 4:**
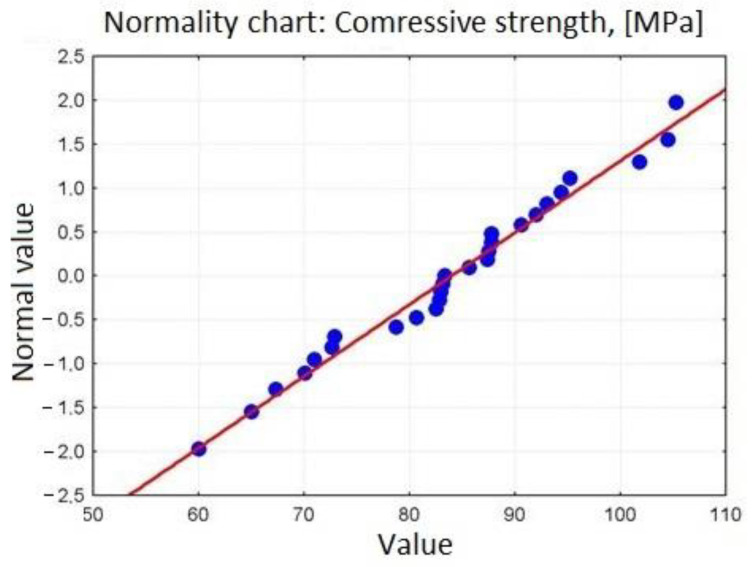
Graph of distribution normality for variables compressive strength.

The chart in Figure 3 suggests that we are dealing with a normal distribution. To check the normal distribution, a diagram of normality for the obtained compressive strength is presented (Figure 4).

The normal distribution hypothesis was verified using the Lilliefors test and the Shapiro–Wilk test (Table 8). It was assumed that:

1. H0: Variable distribution—compressive strength is a normal distribution.
2. H1: Compressive strength is subject to a distribution other than normal.

In both the Lilliefors and Shapiro–Wilk tests, the *p*-value (so-called test probability) is greater than the level of significance of the test, so there is no reason to reject the null hypothesis. Thus, we are dealing with the normal distribution for variables—compressive strength.

### 3.1. ANOVA—Determination of the Effect of Composition on the Compressive Strength of HPSCC

Variable factors for determining and prioritizing the impact on HPSCC compressive strength are w/b ratio, amount of superplasticizer, sand point, and amount of condensed silica fume as well as the amount of paste expressed by the filling’s ratio of the aggregate’s crumb pile with paste, index φ.

Based on the analysis of variance (ANOVA), for the factor systems with the interaction of the obtained test results, the significance of the impact of the tested material factors on the compressive strength of the HPSCC tested was determined.

**Figure 5:**
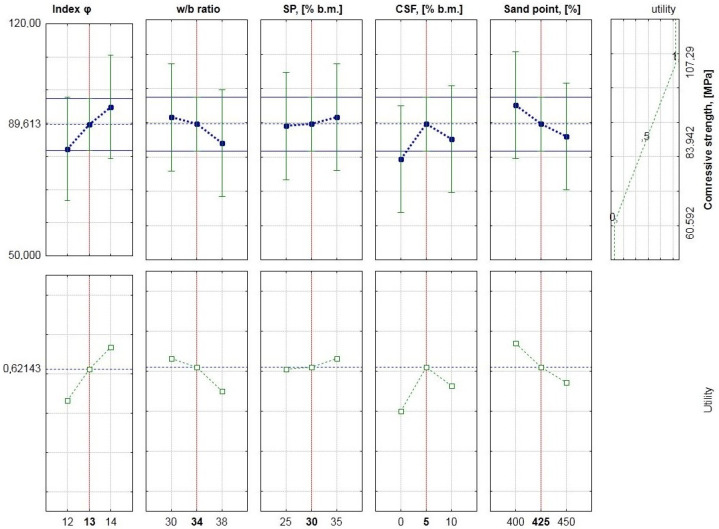
Value profiles of approximated compressive strengths for HPSCC.

The results of the statistical analysis of the significance of the impact of individual factors are presented in Table 9. Variance analysis was performed at a significance level of 0.05 and a confidence interval of 0.95. The value profiles of the approximated compressive strength are shown in Figure 5.

In ordinary concretes, a higher w/c ratio determines better rheological parameters of the mixture but deteriorates compressive strength. For HPSCC concretes, high strength and adequate rheological parameters of the mixture should be ensured at the same time. The rheological properties of SCC are influenced not only by the w/c ratio, but also by the increased amount of binders compared to conventional concretes and the greater amount of fines [87]. These features change not only the rheological properties of the mix, but also the porosity and microstructure in ordinary SCCs compared to conventional concretes, indirectly improving the compressive strength [88]. However, as it turns out from the tests, it is not enough to be able to affect the high strength of HPSCC.

Studies show that the water–binder ratio (w/b) and the amount of condensed silica fume have the greatest impact on compressive strength. The following factors have a smaller impact on the compressive strength, in order: the filling’s ratio of the aggregate’s crumb pile with paste, amount of sand (sand point), and amount of superplasticizer.

The effect of chemical admixture on variability of strength and rheological parameters is negligible, since it is recommended that the dose of superplasticizer should be close to saturation [55,87]. Among the interactions of the examined factors, the water-binder ratio w/b with the amount of condensed silica fume has the greatest impact on the compressive strength.

Increasing the ratio w/b and/or the filling’s ratio of the aggregate’s crumb pile with paste and/or the amount of sand in the form of a sand point causes a reduction in compressive strength. At the same time, increasing the amount of condensed silica fume causes an increase in compressive strength.

### 3.2. Polynomial Regression (Nonlinear Linearized)—Determination of Statistical Models of HPSCC Concrete Compressive Strength and Other Dependencies

**Figure 6:**
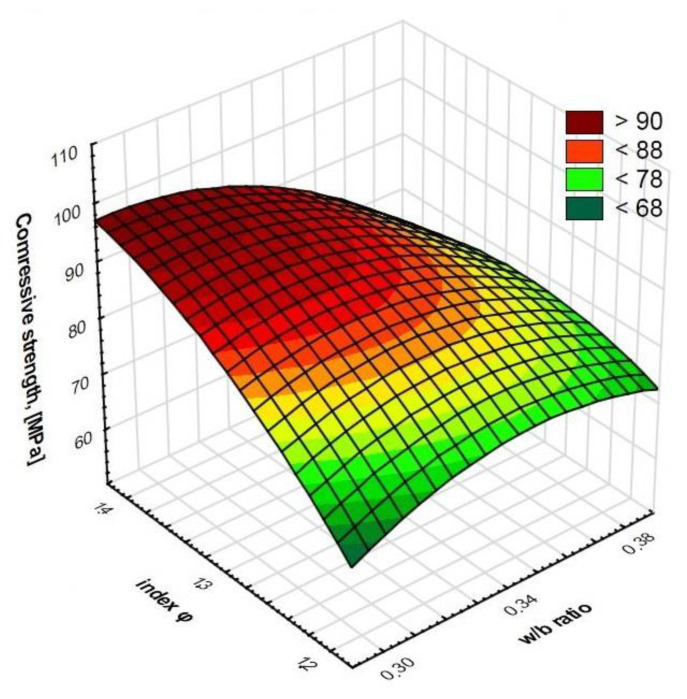
Surface chart. Relationship between w/b ratio and index φ on compressive strength. R^2^ = 0.98.

**Figure 7:**
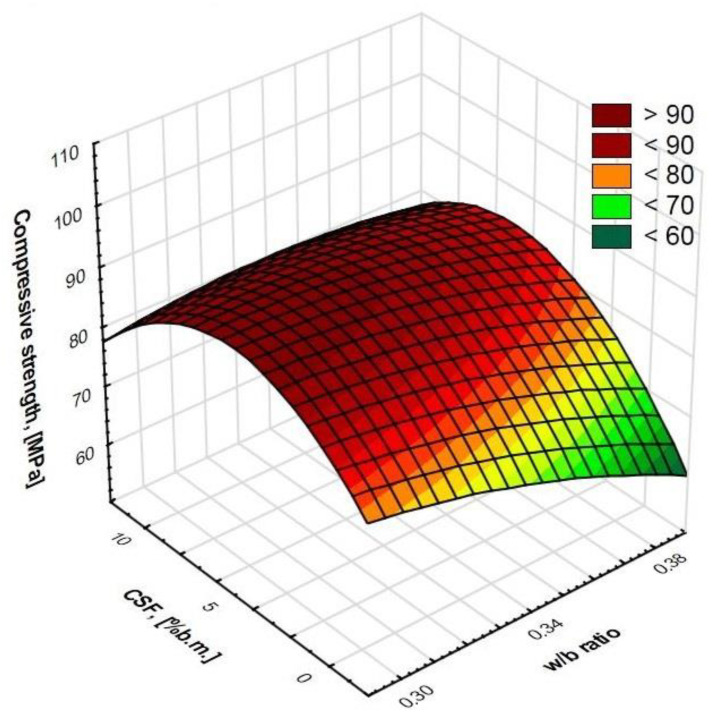
Surface chart. Relationship between w/b ratio and CSF on compressive strength. R^2^ = 0.99.

**Figure 8:**
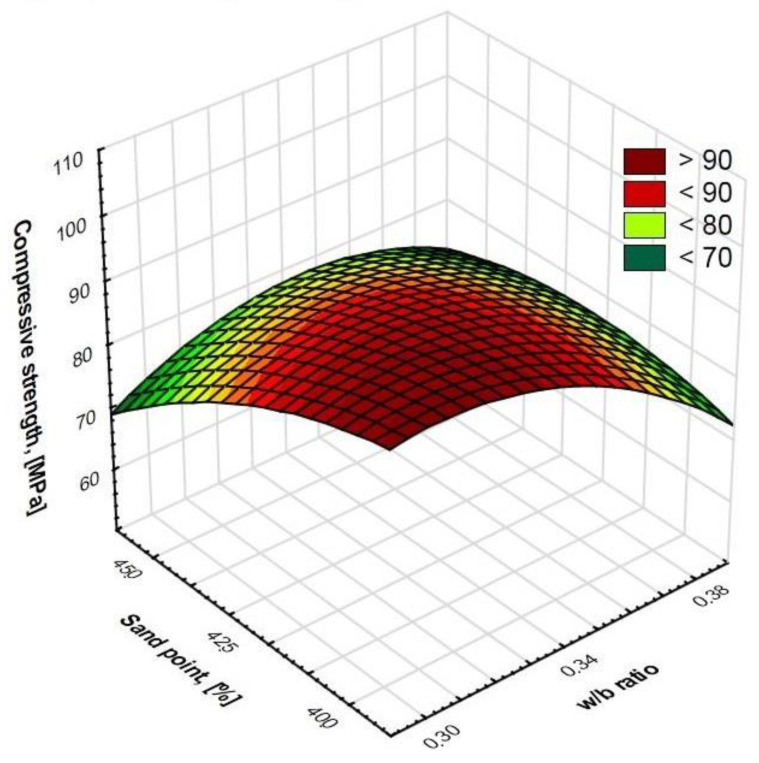
Surface chart. Relationship between w/b ratio and sand point on compressive strength. R^2^ = 0.97.

**Figure 9:**
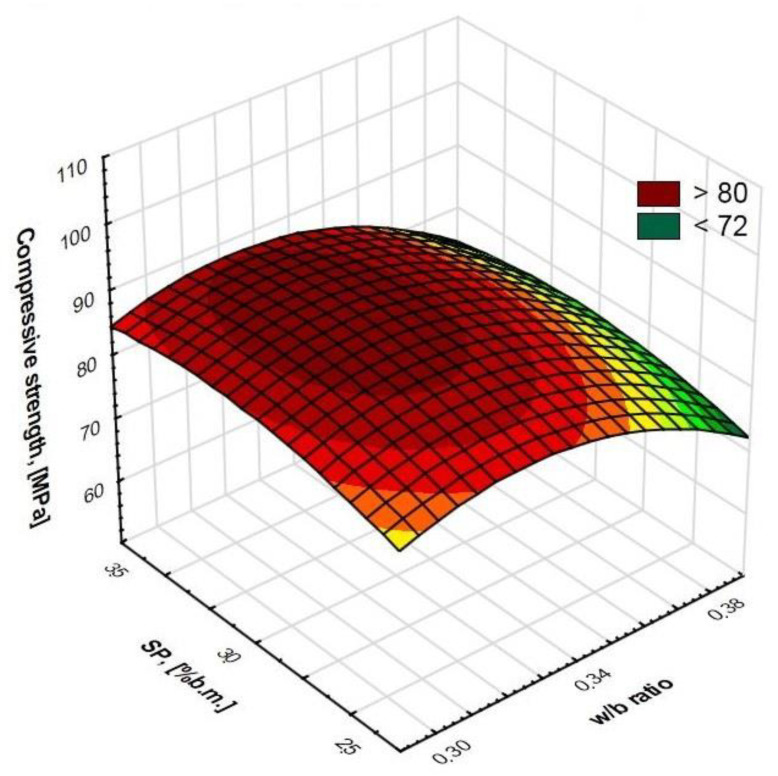
Surface chart. Relationship between w/b ratio and SP on compressive strength. R^2^ = 0.97.

Regression allowed us to describe covariates of the HPSCC composition variables by matching functions to them (Figure 6, Figure 7, Figure 8 and Figure 9). This statistical method allowed us to estimate the conditional expected value of the variable random compressive strength HPSCC for given values of another variable by constructing a regression model, i.e., a function describing how the expected value of the explained variable depends on the explanatory variables. All equations had a high degree of fit, as shown in Figure 6, Figure 7, Figure 8 and Figure 9. Statistical models describing the compressive strength are in the form of second-degree polynomials:

(1) **A**-Index φ; **E**-w/b:

Compressive strength (MPa) = −1333.392 + 31.7232 * **A** + 131.964 * **E** − 0.2551 * **A**^2^ − 1.1783 * **A** * **E** − 3.2909 * **E**^2^; R^2^ = 0.98 (2)

(2) **B**-CSF; **E**-w/b:

Compressive strength (MPa) = 21.6738 + 5.0247 * **B** − 1.133 * **E** − 0.0976 * **B**^2^ + 0.1337 * **B** * **E** − 0.2829 * **E**^2^; R^2^ = 0.99 (3)

(3) **C**-Sand point; **E**-w/b:

Compressive strength (MPa) = −246.832 + 4.6703 * **C** + 1.4632 * **E** − 0.3048 * **C**^2^ + 0.0356 * **C** * **E** − 0.0034 * **E**^2^; R^2^ = 0.97 (4)

(4) **D**-SP; **E**-w/b:

Compressive strength (MPa) = −323.3547 + 19.6374 * **D** + 6.0215 * **E** − 0.2982 * **D**^2^ − 0.0102 * **D** * **E** − 0.0903 * **E**^2^; R^2^ = 0.97 (5)

**Figure 10:**
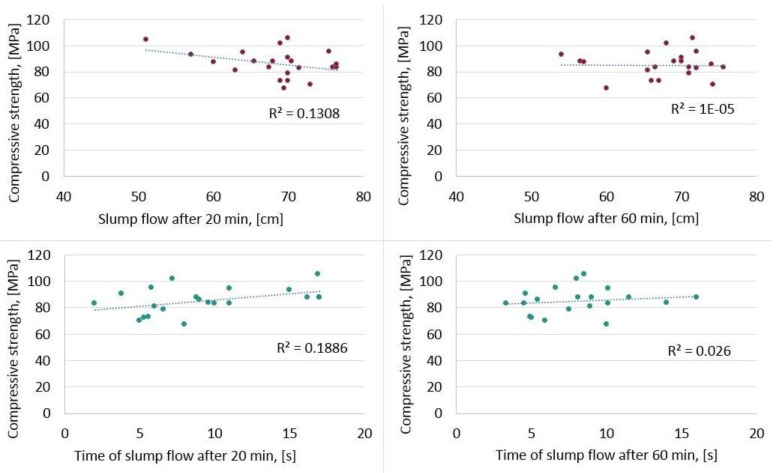
Poor correlation between compressive strength after 28 days and rheological properties: slump flow, D (cm) and time of slump flow, *T* (s).

The self-compacting condition for HPSCC was checked by the standard slump flow test at 20 and 60 min after combining the ingredients. The results allowed the HPSCC concretes to be classified as self-compacting concretes. However, HPSCC should also be designed paying special attention to obtaining the appropriate strength, which was presented in the article. Interestingly, both conditions—self-compacting and the condition of obtaining adequate strength—should be met, but as proved by the research, there is no correlation between them. In Figure 10, the research shows no correlation from a mathematical point of view, because the correlation coefficient is close to zero. There was a weak correlation, i.e., practically no relationship between slump flow and the obtained compressive strength after 28 days and time of slump flow and the obtained compressive strength after 28 days.

In addition to the presented statistical methods—ANOVA, polynomial regression for determination of statistical models of HPSCC concrete compressive strength, and other dependencies—other alternative methods can also be used, e.g., averaging methods to predict elastic properties of pre-impregnated composite materials, as in [89].

## 4. Conclusions and Summary

The tested HPSCC concretes met the characteristics of both self-compacting and high-performance concrete. Its composition was selected after extensive statistical analysis and with great care. The ingredients and the proportions of ingredients used were typical for HPC and SCC concretes. In order to obtain high-quality HPSCC, including high strength, shrinkage, and durability with satisfactory properties of the mixture, the appropriate parameter of diameter of slump flow and slump flow time, the composition of raw materials, chemical and mineral admixtures, aggregate, packing density, and w/b ratio should be should be chosen carefully and planned, which is also confirmed by research [90,91,92,93].

The above-mentioned statistical analysis showed that in 12% of cases, HPSCCs are concretes designed as ordinary SCCs with gravel aggregate, which, however, due to the specificity of the composition ratio, achieved compressive strength ≥ 60 MPa. The other cases in the analysis are concretes already designed as concretes with intentional high strength. Compressive strength is relatively unintentionally often obtained at 60 ÷ 80 MPa, while higher strengths are rarely obtained. Therefore, it is important to be aware of how to shape the compressive strength of HPSCC concrete. This can be helped by the analysis of variance and multiple regression conducted in this article to determine the impact of the universal composition of HPSCC on compressive strength.

After the statistical analysis, the article established:

1. As expected, the water–binder ratio w/b and the amount of condensed silica fume have the greatest impact on compressive strength.
2. Among the interactions of the examined factors, the water–binder ratio w/b with the amount of condensed silica fume has the greatest impact on the compressive strength.
3. Increasing the ratio w/b and/or the filling ratio of the aggregate’s crumb pile with paste and/or the amount of sand in the form of a sand point causes a reduction in compressive strength.
4. At the same time, increasing the amount of condensed silica fume causes an increase in compressive strength.
5. The article also presents statistical models describing the compressive strength using second-degree polynomials with a high degree of fit.
6. Due to the wide range of variability of the basic parameters of HPSCC composition, adjusted to the typical range, used in the research, both the dependencies and functions are general and can be effectively used in supporting HPSCC design. Therefore, the aim of the research was fulfilled.
7. For HPSCC, both conditions—self-compacting and the condition of obtaining adequate strength—should be met, but as proved by the research, there is no correlation between them. There is no relationship between the rheological properties (slump flow and time of slump flow) and the obtained compressive strength after 28 days.

## Figures and Tables

**Table 1:** Analysis of variance of factor tests of the impact of HPSCC composition on 28-day compressive strength [18,21,22,23,24,25,26,27,28,29,30,31,32,33,34,35,36,37,38,39,40,41,42,43,44,45,46,47,48,49,50,51,52,53,54].

Source of Variance	Value F	The Significance Level α
w/b ratio	19.51078	0.006911
Percentage of condensed silica fume, CSF (%)	14.95835	0.046478
Amount of binder, (kg/m^3^)	4.67745	0.082899
Amount of aggregate, (kg/m^3^)	3.09583	0.138813
Amount of cement, (kg/m^3^)	2.36529	0.184673
Amount of sand, (kg/m^3^)	0.05060	0.830927

**Table 2:** Mix design (medians and percentiles) and w/b for concretes: SCC, HPC, and HPSCC.

Ingredients/Ratio	Median	Percentile—10%	Percentile—90%
**Self-compacting concrete (according to P. L. Domone)**			
coarse aggregate (>5 mm) kg/m^3^	895	−	−
sand kg/m^3^	629	−	−
dust fractions (binder < 0.125 mm) kg/m^3^	500	445	605
cement kg/m^3^	−	−	−
water L/m^3^	169	−	−
ratio w/b	0.34	0.28	0.42
**Self-compacting high-performance concrete (own analysis)**			
coarse aggregate (>5 mm) kg/m^3^	1054	910	1190
sand kg/m^3^	700	612	777.5
dust fractions (binder < 0.125 mm) kg/m^3^	486	414	598
cement kg/m^3^	432.5	360	520
water L/m^3^	150	120.1	185.5
ratio w/b	0.31	0.22	0.38
**Self-compaction high-performance concrete (own analysis)**			
coarse aggregate (>5 mm) kg/m^3^	907	698	1027
sand kg/m^3^	742.5	490	910
dust fractions (binder < 0.125 mm) kg/m^3^	560	460	640
cement kg/m^3^	400	350	485
water L/m^3^	167.9	147.9	198
ratio w/b	0.31	0.26	0.40

**Table 3:** Statistics compilation for SCC, HPC and HPSCC aggregate.

	Max. Aggregate Grain Size			Amount of Coarse Aggregate, [kg/m^3^]			Amount of Sand, [kg/m^3^]			Sand Point *, [%]		
	HPSCC	HPC	SCC	HPSCC	HPC	SCC	HPSCC	HPC	SCC	HPSCC	HPC	SCC
median	16	12	20	907	1054	895	742.5	700	629	45	40	−
percentile—25%	14	10	16	793	994.5	−	587	643.5	−	40	38	−
percentile—75%	20	20	20	934	1119	−	884	752	−	53	42	−
average	15	14	18	867.6	1036.8	−	721	705.9	−	45.7	40	−
standard deviation	4.33	4.43	3.4	131.8	143.2	−	173	79.3	−	8	3.6	−

* Sand point is called a percentage of the aggregate mass of particles with dimensions of 0.00 ÷ 2.0 mm (the sum of the percentages of sieve: 0.063, 0.125, 0.25, 0.5, 1).

**Table 4:** Component ranges for composition of HPSCC based on the analysis of literature (analysis of seventy-six compositions) [18,21,22,23,24,25,26,27,28,29,30,31,32,33,34,35,36,37,38,39,40,41,42,43,44,45,46,47,48,49,50,51,52,53,54].

	Minimum	Median	Maximum	Unit
**w/b ratio**	0.25	0.31	0.40	-
**cement**	350	400	500	kg/m^3^
**CSF**	5	9.5	10	%b.m.*
**sand point**	≥40	45	-	%
aggregate	-	**16**	<20	mm
**fraction particulate**	500	560	650	kg/m^3^

* %b.m.—percentage of binder mass (cement + condensed silica fume).

**Table 5:** Table of variable factors in the research in article.

		w/b Ratio	Index φ	SP, %b.m.	CSF, %b.m.	Sand Point, %
Variation in research	The first level	0.30	1.2	2.5	0	40
	The second level	0.34	1.3	3.0	5	42.5
	The third level	0.38	1.4	3.5	10	45

**Table 6:** The research plan [87].

No.	w/b	Index φ	SP (% b.m.)	CSF (% b.m.)	Sand Point (%)
1	0.30	1.2	2.5	0	45
2				10	40
3			3.5	0	40
4				10	45
5		1.3	3.0	5	42.5
6		1.4	2.5	0	40
7				10	45
8			3.5	0	45
9				10	40
10	0.34	1.2	3.0	5	42.5
11		1.3	2.5	5	42.5
12			3.5	5	42.5
13			3.0	0	42.5
14				10	42.5
15				5	40
16				5	45
17 (center)				5	42.5
18		1.4	3.0	5	42.5
19	0.38	1.2	2.5	0	40
20				10	45
21			3.5	0	45
22				10	40
23		1.3	3.0	5	42.5
24		1.4	2.5	0	45
25				10	40
26			3.5	0	40
27				10	45

**Table 7:** Test results.

No.	Visual Stability Index, VSI *	Compressive Strength after 28 Days (MPa)
1	−	60.0
2	−	71.0
3	0	87.8
4	−	65.0
5	0	104.5
6	0	101.8
7	−	92.0
8	1	83.2
9	0	105.3
10	0	93.1
11	0	94.4
12	0	87.8
13	2	78.7
14	0	87.7
15	0	95.2
16	1	87.5
17 (center)	0	83.4
18	1	85.7
19	2	72.9
20	−	82.6
21	2	67.3
22	1	80.7
23	2	72.6
24	2	70.1
25	1	82.9
26	2	83.0
27	1	90.6

* More information in Section 2.3.

**Table 8:** The results of the Lilliefors test and the Shapiro–Wilk test.

Variable	N	Lillief. *p*	S-W	*p*
Compressive strength [MPa]	27	*p* > 0.20	0.976101	0.765805

**Table 9:** Analysis of variance in testing variable factors.

Source of Variance	Compressive Strength	
	Value F	The Significance Level α
**A**: w/b ratio	**7.581**	**0.011909**
**B**: Amount of condensed silica fume, CSF (% b.m.)	2.869	0.134113
**C**: the filling’s ratio of the aggregate’s crumb pile with paste, index φ	1.191	0.311163
**D**: Sand point	0.663	0.442150
**E**: Amount of superplasticizer, SP (% b.m.)	0.001	0.973548
**AB**	**4.566**	**0.093526**
**AC**	2.045	0.235689
**AD**	0.537	0.622562
**AE**	0.245	0.856625
**BC**	0.075	0.792645
**BD**	0.032	0.860248
**BE**	0.063	0.924577
**CD**	0.354	0.554875
**CE**	0.463	0.885454
**DE**	0.253	0.675645

Bold indicates statistically significant impact for significance level α = 0.05.

## Data Availability

The data that support the findings of this study are available from the corresponding authors: (A.K.-S., J.G.) upon reasonable request.

## References

[1] Aïtcin P.-C. (1998). High-Performance Concrete.

[2] Helm M., Hornung F. (1997). Rheological Test Procedure in the Ready-Mixed Concrete Bath Plant. Annu. Trans. Nord. Rheol. Soc..

[3] Neville A.M. (2000). Właściwości Betonu.

[4] Okamura H., Maekawa K., Ozawa K. (1993). High Performance Concrete.

[5] Duval R., Kadri E. H. (1998). Influence of Silica Fume on the Workability and the Compressive Strength of High-Performance Concretes. Cem. Concr. Res..

[6] Okamura H., Ouchi M. Self-Compacting Concrete. Development, Present and Future. Proceedings of the 1st International Symposium on Self-Compacting Concrete.

[7] Mouhcine B.A., Aicha B., Adil H.A., Yves B. (2022). Innovative test for predicting the rheology of self compacting concrete. Mater. Today Proc..

[8] Ioannis P.S., Konstantinos G.T. (2013). Effect of composition variations on bond properties of Self-Compacting, Concrete specimens. Constr. Build. Mater..

[9] Sebaibi N., Benzerzour M., Sebaibi Y., Abriak N.-E. (2013). Composition of self compacting concrete (SCC) using the compressible packing model, the Chinese method and the European standard. Constr. Build. Mater..

[10] Meko B., Ighalo J.O., Ofuyatan O.M. (2021). Enhancement of self-compactability of fresh self-compacting concrete: A review. Clean. Mater..

[11] Ashish D.K., Verma S.K. (2019). Determination of optimum mixture design method for self-compacting concrete: Validation of method with experimental results. Constr. Build. Mater..

[12] Okamura H., Ouchi M. (1998). Self-compacting high performance concrete. Prog. Struct. Eng. Mater..

[13] Okamura H. (1997). Self-Compacting high performance concrete. Concr. Int..

[14] Ozawa K., Maekawa K., Kunishima M., Okamura H. Development of high performance concrete based on the durability design of concrete structures. Proceedings of the Second East-Asia and Pacific Conference on Structural Engineering and Construction (EASEC-2).

[15] Sari M., Prat E., Labastire J.-F. (1999). High strength self-compacting concrete Original solutions associating organic and inorganic admixtures. Cem. Concr. Res..

[16] (2007). ACI 237R-07. Self-Consolidating Concrete.

[17] Benaicha M., Belcaid A., Alaoui A.H., Jalbaud O., Burtschell Y. (2019). Rheological characterization of self-compacting concrete: New recommendation. Struct. Concr..

[18] De Schutter G., Bartos P.J.M., Domone P., Gibbs J. (2008). Self Compacting Concrete.

[19] SCC European Project Group (2005). EFNARC: The European Guidelines For Self-Compacting Concrete. Specif. Prod. Use.

[20] Kashani A., Ngo T. (2020). 3: Production and placement of self-compacting concrete. Self-Compact. Concr. Mater. Prop. Appl..

[21] Wallevik O., Nielsson I. Self-Compacting Concrete. Proceedings of the Third International RILEM Symposium, RILEM Proceedings PRO 33.

[22] Kostrzanowska-Siedlarz A., Gołaszewski J. (2015). Rheological properties and the air content in fresh concrete for self compacting high performance concrete. Constr. Build. Mater..

[23] Jalal M., Pouladkhan A., Fasihi Harandi O., Jafari D. (2015). Comparative study on effects of Class F fly ash, nano silica and silica fume on properties of high performance self compacting concrete. Constr. Build. Mater..

[24] Collepardi M., Collepardi S., Ogoumah Olagot J.J., Troli R. Laboratory tests and field experiences of high performance SCCs. Proceedings of the 3rd International Symposium on Self-Compacting Concrete.

[25] Dybeł P., Kucharska M. (2020). Effect of bottom-up placing on bond properties of high-performance self-compacting concrete. Constr. Build. Mater..

[26] Lu C., Yang H., Mei G. (2015). Relationship between slump flow and rheological properties of self compacting concrete with silica fume and its permeability. Constr. Build. Mater..

[27] Ding Y., Zhang Y., Thomas A. (2009). The investigation on strength and flexural toughness of fibre cocktail reinforced self-compacting high performance concrete. Constr. Build. Mater..

[28] Li J., Yin J., Zhou S., Li Y. Mix proportion calculation method of self-compacting high performance concrete. Proceedings of the First International Symposium on Design, Performance and Use of Self-Consolidating SCC’2005-China.

[29] Ozbay E., Oztas A., Baykasoglu A., Ozbebek H. (2009). Investigating mix proportions of high strength self compacting concrete by using Taguchi method. Constr. Build. Mater..

[30] Rougeau P., Maillard J.L., Mary C.-D. Comparative study on properties of self-compacting and high performance concrete used in precast construction. Proceedings of the International RILEM Symposium on Self-Compacting Concrete No1.

[31] Matos A.M., Maia L., Nunes S., Milheiro-Oliveira P. (2018). Design of self-compacting high-performance concrete: Study of mortar phase. Constr. Build. Mater..

[32] Ravindrarajah R.S., Siladyi D., Adamopoulos B. Development of High-Strengh Self-Compacting Concrete with reduced segregation potential. Proceedings of the 3rd International Symposium on Self-Compacting Concrete.

[33] Tang C.W., Yen T., Chang C.S., Chen K.H. (2001). Optimizing mixture for flowable high-performance concrete via rheology tests. ACI Mater. J..

[34] Shi C., Wu Z., Lv K., Wu L. (2015). A review on mixture design methods for self-compacting concrete. Constr. Build. Mater..

[35] Xie Y., Liu B., Yin J., Zhou S. (2002). Optimum mix parameters of HSSCC with ultrapulverized fly ash. Cem. Concr. Res. V.

[36] Yin J., Xie Y., Yu Z. Optimization of fabrication technology of Self-Compacting High Performance Concrete. Proceedings of the First International Symposium on Design, Performance and Use of Self-Consolidating SCC’2005-China.

[37] Eskandari H., Raghu Prasad B.K., Venkatarama Reddy B.V. (2009). Prediction of compressive strength of SCC and HPC with high volume fly ash using ANN. Constr. Build. Mater..

[38] Akalin O., Akay K.U., Sennaroglu B. Statistical approach to high strength concrete mixture proportioning. Proceedings of the Tenth ACI International Conference on Recent Advances in Concrete Technology and Sustainability Issues.

[39] Matos P.R., Sakata R.D., Prudêncio L.R. (2019). Eco-efficient low binder high-performance self-compacting concretes. Constr. Build. Mater..

[40] Paulou K. Pre-testing of self-compacting concrete with various mineral additives and admixtures. Proceedings of the 3rd International Symposium on Self-Compacting Concrete.

[41] Turcry P., Loukili A. A study of plastic shrinkage of self-compacting concrete. Proceedings of the 3rd International Symposium on Self-Compacting Concrete.

[42] Khayat K.H., Petrov N., Attiogbe E.K., See H.T. Uniformity of bond strength of prestressing strands in conventional flowable and self-consolidating concrete mixtures. Proceedings of the 3rd International Symposium on Self-Compacting Concrete.

[43] Fredvik T.I., Gundersen N.L., Johansen K. Development of SCC in Norway-use of CSF. Proceedings of the 3rd International Symposium on Self-Compacting Concrete.

[44] Zhu W., Bartos P. Microstructure and properties of interfacial transition zone in SCC. Proceedings of the First International Symposium on Design, Performance and Use of Self-Consolidating SCC’2005-China.

[45] Poppe A.M., De Schutter G. Creep and shrinkage of self-compacting concrete. Proceedings of the First International Symposium on Design, Performance and Use of Self-Consolidating SCC’2005-China.

[46] Audenaert K., Boel V., De Schutter G. Chloride penetration in self-compacting concrete by cyclic immersion, Performance and Use of Self-Consolidating SCC’2005-China. Proceedings of the 5th International RILEM Symposium on Self-Compacting Concrete.

[47] Assie S., Escadeillas G., Waller V. (2007). Estimates of self-compacting concrete ‘potential’ durability. Constr. Build. Mater..

[48] Persson B. (2001). A comparison between mechanical properties of self-compacting concrete and the corresponding properties of normal concrete. Cem. Concr. Res..

[49] Le H.T., Müller M., Siewert K., Ludwig H.-M. (2015). The mix design for self-compacting high performance concrete containing various mineral admixtures. Mater. Des..

[50] Gesoğlu M., Güneyisi E., Özbay E. (2009). Properties of self-compacting concretes made with binary, ternary and quaternary cementitious blends of fly ash, blast furnace slag, and silica fume. Constr. Build. Mater..

[51] Skarendahl A., Billberg P. (2006). Casting of Self Compacting Concrete, RILEM Report 35.

[52] Skarendahl Å., Petersson Ö. Self-Compacting Concrete. Proceedings of the First International RILEM Symposium, RILEM Proceedings 7.

[53] Szwabowski J., Banfill P.F.G. (1991). Influence of three-phase structure on the yield stress of fresh concrete, Rheology of fresh cement and concrete. Proceedings of the International Conference organized by the British Society of Rheology, University of Liverpool, University of Liverpool.

[54] Domone P.J., Jin J. Properties of mortar for self-compacting concrete. Proceedings of the Self-Compacting Concrete.

[55] Domone P.L. (2006). Self-compacting concrete: An analysis of 11 years of case studies. Cem. Concr. Compos..

[56] Nguyen N.-H., Vo T. P., Lee S., Asteris P.A. (2021). Heuristic algorithm-based semi-empirical formulas for estimating the compressive strength of the normal and high performance concrete. Constr. Build. Mater..

[57] Al-Jabri K.S., Hisada M., Al-Saidy A.H., Al-Oraimi S.K. (2009). Performance of high strength concrete made with copper slag as a fine aggregate. Constr. Build. Mater..

[58] Aïtcin P.-C. (1998). High-Performance Concrete.

[59] Afroughsabet V., Teng S. (2020). Experiments on drying shrinkage and creep of high performance hybrid-fiber-reinforced concrete. Cem. Concr. Compos..

[60] Akhnoukh A.K. (2019). Accelerated bridge construction projects using high performance concrete. Case Stud. Constr. Mater..

[61] Ma J., Dietz J. (2002). Ultra High Performance Compating Concrete. Lacer.

[62] Zain M.F.M., Islam M.N., Basri I.H. (2005). An expert system for mix design of high performance concrete. Adv. Eng. Softw..

[63] Persson B. (1996). Hydration and Strength of High Performance Concrete. Adv. Cem. Based Mater..

[64] Brandt A.M. (2005). Cement-Based Composites. Materials, Mechanical Properties and Performance.

[65] Nagamoto N., Ozawa K. (1999). Mixture proportions of self-compacting high performance concrete. ACI Int..

[66] ACI Committee 226 (1987). Silica Fume in Concrete. ACI Mater. J..

[67] Telford T. (1998). Condensed Silica Fume in Concrete. State of Art Report FIB/CEB Bulletin D’information.

[68] Jasiczak J., Mikołajczyk P. (1997). Technologia betonu modyfikowanego domieszkami i dodatkami. Przegląd tendencji krajowych i zagranicznych. Wydawnictwa Politechniki Poznańskiej, Technology of Concrete Modified with Admixtures and Additions.

[69] Parichatprecha R., Nimityongskul P. (2009). Analysis of durability of high performance concrete using artificial neural networks. Constr. Build. Mater..

[70] Hamid R., Yusof K.M., Zain M.F.M. (2010). A combined ultrasound method applied to high performance concrete with silica fume. Constr. Build. Mater..

[71] Horszczaruk E. (2008). Mathematical model of abrasive wear of high performance concrete. Wear.

[72] Hossain K.M.A. (2006). High strength blended cement concrete incorporating volcanic ash: Performance at high temperatures. Cem. Concr. Compos..

[73] Kadri E.-H., Aggoun S., De Schutter G. (2009). Interaction between C_3_A, silica fume and naphthalene sulphonate superplasticiser in high performance concrete. Constr. Build. Mater..

[74] Picandet V., Bastian G., Khelidj A. (2008). Compared imbibitions of ordinary and high performance concrete with null or positive water pressure head. Cem. Concr. Res..

[75] Mustapha F.A., Sulaiman A., Mohamed R.N., Umara S.A. (2021). The effect of fly ash and silica fume on self-compacting high-performance concrete. Mater. Today Proc..

[76] Zain M.F.M., Safiuddin M., Yusof K.M. (1999). A study on the properties of freshly mixed high performance concrete. Cem. Concr. Res..

[77] Giergiczny Z., Małolepszy J., Szwabowski J., Śliwinski J. (2002). Cementy z dodatkami mineralnymi w technologii betonów nowej generacji. Wydawnictwo Instytut Śląski, Cements with Mineral Additives in the New Generation Concrete Technology.

[78] Khayat K.H., Saric-Coric M., Tagnit-Hamou A. (2003). Performance characteristics of cement grouts made with various combinations of high-range water reducer and cellulose-based viscosity modifier. Cem. Concr. Res..

[79] Khayat K.H. International RILEM Symposium of Self-Compacting Concrete. Proceedings of the Design, Production and Placement of SCC.

[80] Gołaszewski J. (2009). Influence of Viscosity Enhancing Agent on Rheology and Compressive Strength of Superplasticized Mortars. J. Civ. Eng. Manag..

[81] Łaźniewska-Piekarczyk B. (2011). Wpływ domieszek stabilizujących lepkość (DSL) na właściwości samozagęszczających się zapraw i betonów. Cem. Wapno Beton.

[82] Łaźniewska-Piekarczyk B. (2013). Effect of viscosity type modifying admixture on porosity, compressive strength and water penetration of high performance self-compacting concrete. Constr. Build. Mater..

[83] Gutierrez P.A., Canowas M.F. (1996). High-Performance Concrete: Requirements for Constituent Materials and Mix Proportioning. ACI Mater. J..

[84] Kosmatka S., Kerkhoff B., Panarese W. (2000). High Performance Concrete. Design and Control of Concrete Mixtures.

[85] Carlsward J., Emborg M., Utsi S., Oberg P. Effect of constituents on the workability and rheology of self-compacting concrete. Proceedings of the 3rd International Symposium on Self-Compacting Concrete.

[86] Szwabowski J., Łaźniewska-Piekarczy B. (2008). Zwiększenie napowietrzenia mieszanki pod wpływem działania superplastyfikatorów karboksylowych, Increasing the aeration of the mixture under the influence of carboxylic superplasticizers. Cem. Wapno Beton.

[87] Kostrzanowska-Siedlarz A., Gołaszewski J. (2016). Rheological properties of High Performance Self-Compacting Concrete:Effects of composition and time. Constr. Build. Mater..

[88] Wallevik O. (2002). Course on Rheology-Rheology of Cement Suspensions.

[89] Teodorescu-Draghicescu H., Vlase S. (2011). Homogenization and averaging methods to predict elastic properties of pre-impregnated composite materials. Comput. Mater. Sci..

[90] Han J., Fang H., Wang K. (2014). Design and control shrinkage behavior of high-strength self-consolidating concrete using shrinkage-reducing admixture and super-absorbent polymer. J. Sustain. Cem. Based Mater..

[91] Esmaeilkhanian B., Khayat K.H., Yahia A., Feys D. (2014). Effects of mix design parameters and rheological properties on dynamic stability of self-consolidating concrete. Cem. Concr. Compos..

[92] Siddique R., Aggarwal P., Aggarwal Y. (2012). Mechanical and durability properties of self-compacting concrete containing fly ash and bottom ash. J. Sustain. Cem. Based Mater..

[93] Wang X.H., Wang K.J., Taylor P., Morcous G. (2014). Assessing particle packing based self-consolidating concrete mix design method. Constr. Build Mater..

